# One Health assessment of zoonotic intestinal parasites in humans, dogs, and soil of coastal Cartagena, Colombia

**DOI:** 10.14202/vetworld.2025.3352-3366

**Published:** 2025-11-06

**Authors:** Dilia Mildret Fontalvo Rivera, Irina Tirado Ballestas, Marina Morales Aleans, Javier Moreno Meneses, Natalia Lemos Calle, Mariana Lucía Mier Fontalvo, Sofía Plata Suarez, Anyel Bertel De La Hoz, Javier Galvis Ballesteros, Vanessa Quiñones Cantillo, Jimmy Piñeros Passos, Luis Conde Berrío, Nuria Visbal Giraldo, Camila Carbal Carvajal, Andrea Santos Muñoz, Juan Franco Rodríguez, Alejanddro Hurtado Martínez, Karen Navarro González, Daniela Sierra Urueta, María Lina Simancas Mogollón

**Affiliations:** 1 Department of Medicine and Research, Faculty of Medicine, Universidad del Sinú, Cartagena, Colombia; 2 Microbiology Laboratory, Faculty of Basic Sciences, Universidad del Sinú, Cartagena, Colombia; 3 Microbiology Laboratory, Microbiology and Environment Research Group – GIMA, Cartagena, Colombia; 4 Veterinary Study Program, Faculty of Veterinary Medicine, Universidad de Ciencias Ambientales y Aplicadas, UDCA, Cartagena, Colombia

**Keywords:** canines, Cartagena, Intestinal parasitosis, One Health, soil contamination, zoonoses

## Abstract

**Background and Aim:**

Zoonotic intestinal parasites remain a neglected public health problem in low-resource coastal communities where humans, free-roaming dogs, and contaminated environments interact closely. Cartagena, Colombia, lacks updated epidemiological data on intestinal parasitosis despite its high tourist influx and vulnerable populations. This study applied a One Health framework to investigate the prevalence of intestinal parasites in humans, dogs, and soil in two coastal regions of Cartagena (La Boquilla and Punta Arenas) and to identify associated risk factors and clinical manifestations.

**Materials and Methods:**

A cross-sectional survey was conducted between March 2024 and March 2025. Stool samples from 33 residents and 42 dogs were analyzed by direct microscopy with saline and Lugol’s solutions, while 78 soil samples were assessed using the Krumbein, Sloss, and Willis techniques. Dogs suspected of *Dirofilaria* spp. infection were additionally screened by the Woo test. Sociodemographic data, nutritional status, and clinical information were collected. Statistical analyses included descriptive tests, Chi-square/Fisher’s tests, logistic regression, and odds ratio (OR) estimation at a significance level of p ≤ 0.05.

**Results:**

Soil samples showed high contamination with *Toxocara* spp. (46.2%), *Strongyloides* spp. (28%), and *Ancylostoma* spp. (25.7%). Among humans, 60.97% were positive for parasites, with *Giardia* spp. (15.15%), *Entamoeba histolytica/dispar* (12.12%), *Ascaris lumbricoides* (12.12%), and *Enterobius vermicularis* (12.12%) being the most frequent. In dogs, 33.33% carried intestinal parasites, predominantly *Ancylostoma* spp. (14.29%) and *Giardia* spp. (7.14%). Clinical manifestations in humans included loss of appetite, cough, dermatitis, and weight loss, while dogs frequently presented with pallor, dermatological lesions, and gastrointestinal signs. Logistic regression analysis indicated that a lack of canine deworming significantly increased the risk of human parasitic infections (OR: 3.80; 95% confidence interval: 0.98–14.66; p = 0.048).

**Conclusion:**

This One Health investigation highlights significant zoonotic risk from shared parasitic infections in humans, dogs, and contaminated soils in Cartagena’s coastal regions. The lack of systematic deworming and vaccination in dogs, poor sanitation, and close human–dog contact amplify transmission. Strengths of this study include its integrative human–animal–environment approach, while limitations involve modest sample size and lack of molecular genotyping. Future work should apply molecular epidemiology to confirm cross-species transmission. Practical implications emphasize the urgent need for mass deworming campaigns, improved waste management, vector control, and public health education to reduce zoonotic intestinal parasite burden in vulnerable coastal communities.

## INTRODUCTION

Companion animals, particularly dogs and cats, have become integral members of human society. Increasingly, families adopt pets not only for companionship but also for their role in providing emotional support during recovery from illness. However, this close bond has heightened the risk of transmission of parasitic zoonoses. Notable helminths include *Ancylostoma caninum*, *Trichuris vulpis*, *Strongyloides stercoralis*, *Dipylidium caninum*, *Toxocara canis*, and *Taenia crassiceps*, while protozoa such as *Giardia duodenalis* and *Cryptosporidium* spp. are also of concern [1, 2]. Zoonoses pose a significant global public health challenge, imposing a substantial economic burden on healthcare systems. Their incidence continues to rise, driven by social, economic, and cultural factors such as globalization, migration, and the displacement of humans and animals. Globally, it is estimated that 35% of zoonotic diseases are parasitic in origin [3].

In Colombia, the control of parasitic zoonoses remains inadequate due to limited resources and insufficient public health surveillance. For example, the most recent official report (2022) from Bogotá indicated that dogs accounted for the majority of notifications to the National Public Health Surveillance System (Sivigila), representing 89.7% (602/671 cases). Within these, 4.5% (27/671) were attributed to pathogens, such as bacteria, intestinal protozoa, and parasites. Specifically, *Campylobacter*, *Giardia*, *Coccidia*, and *Cryptosporidium* were reported by veterinary clinics, underscoring their relevance as zoonotic threats transmitted through contaminated food, water, or inadequate hygiene practices, such as failing to wash hands after handling animals or their feces [1].

Although parasitic zoonoses are widely recognized as a global health burden, there is a lack of updated epidemiological data from many low- and middle-income countries, including Colombia. Most national reports remain fragmented, with limited integration of human, animal, and environmental health perspectives. In Bogotá, recent surveillance data showed that canines contributed the majority of zoonotic disease notifications, yet these records largely reflected bacterial and protozoal pathogens without comprehensive characterization of intestinal parasites across species. Moreover, surveillance efforts are heavily concentrated in major urban centers, leaving peripheral and coastal regions such as Cartagena underrepresented. Despite being a high-traffic tourist destination with unique ecological and socioeconomic conditions that favor parasite transmission, such as free-roaming dogs, poor waste management, and limited access to sanitation, Cartagena lacks official data on zoonotic intestinal parasites in humans, companion animals, or the environment. Additionally, few studies in Colombia have adopted the One Health approach, which is essential to understand the interconnected risks posed by shared habitats and interactions among humans, animals, and contaminated soils. This creates a critical gap in knowledge regarding the epidemiology, risk factors, and clinical impact of intestinal parasites in vulnerable coastal communities.

To address this gap, the present study employed a One Health framework to investigate zoonotic intestinal parasites in two coastal regions of Cartagena, Colombia (La Boquilla and Punta Arenas). Specifically, the study aimed to (i) determine the prevalence and diversity of intestinal parasites in humans, dogs, and soil samples; (ii) assess sociodemographic, clinical, and environmental factors associated with parasite transmission; and (iii) identify shared parasitic species between humans and dogs that could indicate cross-species transmission. By integrating data from human health, veterinary health, and environmental sampling, this research provides the first comprehensive evaluation of zoonotic intestinal parasitoses in Cartagena’s coastal areas. The findings are expected to inform targeted interventions such as mass deworming campaigns, improved sanitation, and public health education programs, ultimately contributing to the reduction of zoonotic risks in both local communities and visiting populations.

## MATERIALS AND METHODS

### Ethical approval and Informed consent

The study adhered to the principles outlined in Resolution 8430 of 1993 and Resolution 2378 of 2008 regarding Good Clinical Practices. Informed consent was obtained from parents/guardians of minors and dog owners. Ethical principles included confidentiality of personal data, voluntary participation, and no use of drugs or unapproved diagnostic methods.

The study was approved by the Bioethics Committee of the Universidad del Sinú, Cartagena, on December 21, 2023 (Code MED-PD/2024-), under Rectoral Resolution #037 of November 22, 2018. It complied with Colombian legislation on animal welfare (Law 1955/2019, Article 3241) and rulings of the Constitutional Court (C-041/2017, C-283/2014, T-095/2016).

### Study period and location

The study was conducted between March 2024 and March 2025 in La Boquilla and Punta Arenas, Tierra Bomba Island, Cartagena, Colombia. Punta Arenas has a population density of 1,850 inhabitants/km² and is only accessible by boat. Ninety-six percent of the homes in the township require housing improvements and lack access to potable water and sewerage services. Waste management is performed using mopeds [4].

**Figure 1:**
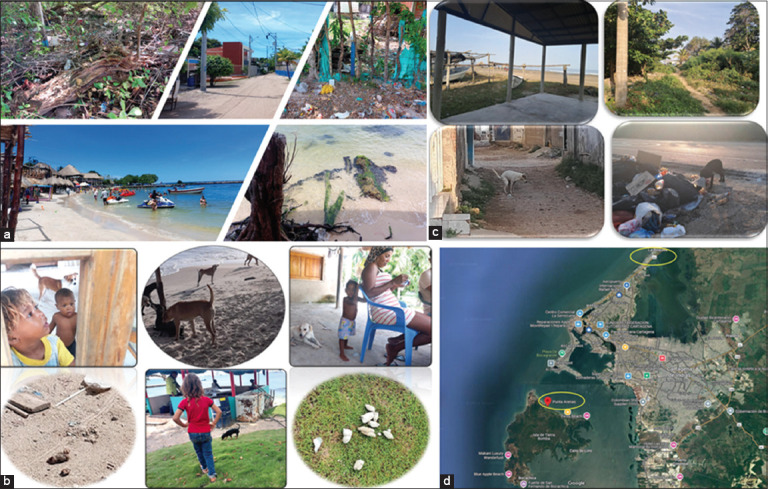
Biodiversity in the coastal regions of Punta Arenas (Tierra Bomba Island) and La Boquilla, Cartagena, Colombia. (a) In Punta Arenas, the sea landscapes, beaches with coral reefs, and rich vegetation contrast with areas ofwaste contamination. (b) Dog feces that move freely through public spaces. (c) The coastal area of La Boquilla is framed by maritime tradition, with areas of free transit associated with zoonotic risk. (d) The soil study was conducted in the coastal areas of La Boquilla and Punta Arenas (yellow ovals) in Cartagena, Colombia.

The ecosystem is diverse, with a tropical dry forest of 998,709 hectares (ha), a mangrove forest of 257,909 ha, and extensive beaches, making tourism one of the main economic activities [5, 6]. Landscapes such as white sand beaches, turquoise waters, and lush green vegetation contrast with dilapidated houses and poor environmental health conditions. Weed infestations occur in areas with accumulated debris, favoring the spread of pathogens that cause infectious and respiratory diseases (Figure 1a).

On the roads and in some dwellings, canines of various breeds roam freely, often accompanied by their owners, who report that dogs defecate on public roads, parks, and scrub areas frequently used by local residents (Figure 1b).

La Boquilla also has a rich diversity of natural resources due to its varied ecosystems. To the east, it borders Ciénaga de La Virgen with extensive mangroves and diverse fauna. To the west, it connects with the Caribbean Sea, and to the north, it is bordered by tropical dry forest. The township has approximately 25,000 inhabitants, and tourism-related activities are common along the beaches [7]. Waste is often placed on road corners for collection by cleaning services, where animals scavenge, and dogs defecate freely in public spaces (Figure 1c).

### Study design and sample size

This was a descriptive study that used non-probabilistic (convenience) sampling to include the largest possible number of willing participants. This approach was chosen because it was the first study of its kind in this geographic region, simultaneously involving the three axes of health: human, animal, and environmental.

The target population included humans and dogs of all ages. Puppies were considered to be dogs up to 1 year of age, young adults from 1 to 4 years of age, adults from 5 to 8 years of age, and seniors older than 9 years of age. The dogs’ nutritional status was determined according to the Canine Body Condition Index of the Global Nutrition Committee Toolkit [8].

The monetary income of the human participants was classified according to the socioeconomic strata system in Law 142 of 1994 of the Colombian Constitution: level 1 (low-low), 2 (low), 3 (medium-low), 4 (medium), 5 (medium-high), and 6 (high) [9].

The humans and dogs included in the study did not have to belong to the same household; however, they needed to have direct interaction. Human participants with chronic gastrointestinal conditions, such as malabsorption disorders or recent anthelmintic use, were excluded. All dogs, whether owned or free-roaming, and regardless of vaccination or deworming status, were included.

### One Health working group

To ensure an integrative approach, interinstitutional working groups were established covering animal health (Figure S1a), environmental health and microbiology (Figure S1b), and human health (Figure S1c), in alignment with the WHO One Health objectives.

### Sampling in humans and dogs

Before collecting samples, all participants were instructed on collection techniques (Figures S1d, S2a and b).

#### Canine sampling

Clinical evaluations were carried out by veterinary professors, researchers, and students. Dogs were restrained with the assistance of their owners and veterinary staff members. After aseptic preparation of the venipuncture site, 2 mL of blood was collected from small breeds and 4 mL from medium and large breeds, using ethylenediaminetetraacetate tubes with lilac-colored caps. Blood samples were transported at 4°C–6°C to the laboratory on the same day. Dogs with clinical signs of endothelial damage or vasculitis were tested for *Dirofilaria* spp. using the Woo hematocrit centrifugation technique [10].

#### Human and canine stool sampling

One stool sample was collected from each participant. Fresh canine feces were collected immediately after defecation (approximately two to four beans in size) in sterile containers. Human participants were advised not to consume red meat the day before sampling, and samples were collected under sterile conditions using spatulas. All fecal samples were stored in coolers at 4°C–6°C and transported to the Universidad del Sinú laboratory in Cartagena for analysis (Figure S3).

### Soil sampling

Soil samples were collected at sites with high human and canine activity in La Boquilla and Punta Arenas (Figure 1c). Sampling points were selected based on the INCAPTU-RG index [11, 12].

For Punta Arenas, samples were collected at coordinates 10°21′57.30″ N, 75°33′4.8″ W (point 1). For La Boquilla, samples were taken from points at 10°24′2.4″ N, 75°33′16.4″ W (P1); 10°27′37.05″ N, 75°30′22.66″ W (point 2); 10°28′15.17″ N, 75°29′54.82″ W (point 3); and 10°28′59.29″ N, 75°29′32.98″ W (point 4) (Figure 1d).

The region has a tropical climate with two main seasons: a dry season (December–April) and a rainy season (May–November), with peak rainfall in September–October. Climate change in Cartagena, including rising sea levels, coastal erosion, and saltwater intrusion, complicates predictions of parasite distribution. To reduce bias, monthly sampling was performed during both the dry and rainy seasons, with sand collected in the morning and afternoon at all points. Approximately 200 g of sand was collected at 10 cm depth from the active beach zones, placed in Ziploc bags, labeled (date, time, location, and collector’s name), and transported for laboratory analysis.

### Parasitological analysis of human and canine samples

Fecal samples were first subjected to macroscopic examination [13], followed by microscopic evaluation using Lugol’s solution (5 g iodine, 10 g potassium iodide in 85 mL distilled water) [14] and 0.9% saline solution [15, 16]. Analyses were conducted independently by three team members (a microbiologist, an environmental parasitologist, and a veterinary parasitologist) at the Universidad del Sinú laboratory, which is accredited under Colombian Ministry of Health Resolution 3100 of 2019.

### Microbiological analysis of soil samples

Soil samples were analyzed using Krumbein, Sloss, and Willis techniques. The Willis flotation method concentrated parasitic forms (protozoan cysts and helminth eggs) using saturated sodium chloride. The Sloss flotation–sedimentation method with Sheather’s reagent was used to identify helminth eggs and larvae [17, 18]. Morphological features, such as size, shape, shell thickness, opercula, spines, and internal embryos were used for species identification.

### Statistical analysis

- Nominal variables were reported as frequencies and percentages, with 95% confidence intervals (CIs)
- Continuous data were expressed as means ± standard deviation (SD) or medians with interquartile ranges, depending on normality (Shapiro–Wilk test)
- Associations between categorical variables were tested with chi-square or Fisher’s exact test
- Comparisons of means were performed with Student’s two paired t-test, two-way analysis of Variance, Bartlett test, or Mann–Whitney test, depending on distribution.
- Logistic regression (cumulative logit model) was used to identify risk factors associated with human parasitic infections. Predictor variables included sex, nutritional status, barefoot exposure (in humans), free-roaming behavior, breed, and deworming history (in dogs). Odds ratios (OR) with 95% CIs were calculated. The proportionality assumption of the model was tested (p = 0.7469), confirming model suitability.
- Statistical analyses were conducted using SAS 9.4, STATA 15, and Epi Info 7. Geographic maps were generated with Google Maps, and figures were produced in Epi Info 7 and Microsoft Excel.

## RESULTS

### Environmental axis: Soil contamination with canine intestinal parasites

#### Soil sampling and overall positivity

In this study, 78 samples were collected from two beaches: 48 from La Boquilla and 30 from Punta Arena. A total of 50.7% of these samples were positive for one or more forms of the gastrointestinal parasites *Toxocara* spp., *Ancylostoma* spp., and *Strongyloides* spp., revealing a total of 171 infective forms. The highest percentage of parasites corresponded to *Toxocara* spp. (79 [46.2%]), followed by *Strongyloides* spp. (48 [28%]) and *Ancylostoma* spp. (44 [25.7%]) (Table 1).

**Table 1:** Percentage of intestinal parasites in the soils of the sectors analyzed.

Genus of the parasites	N (%)	Punta Arenas Beach n (%)	La Boquilla Beach, Mexico n (%)		

			**Bo1	Bo2	Bo3
*Strongyloides* spp.	48 (28)	7 (58.3)	24 (15.1)	10 (6.3)	7 (4.4)
*Ancylostoma* spp. eggs	44 (25.7)	1 (8.3)	28 (17.6)	7 (4.4)	8 (5.0)
*Toxocara* spp. eggs	79 (46.2)	4 (33.3)	21 (13.2)	39 (24.5)	15 (9.4)
Total	171 (100)	12 (100)	73 (45.9)	56 (35.2)	30 (18.9)

** Bo: La Boquilla. Significant contamination by intestinal parasites was detected in soil samples collected from public transit areas in the analyzed sectors. Larvae and embryonated eggs with the ability to infect other species were present in the samples.

#### Seasonality

Climate conditions can influence parasite growth [19, 20]. In this study, the abundance of *Strongyloides* and *Toxocara* spp. in the beach sand samples was greater in the dry season (14.1% and 30.9%, respectively), and the parasite genus *Ancylostoma* spp. abundance was greater in the rainy season (14.8%).

### Human health axis

#### Sociodemographic characteristics

The population that met the estimated eligibility criteria included 104 residents who had contact with free-roaming dogs or with feces on public roads; 54 were excluded because they did not provide informed consent, and 17 did not provide fecal samples, leaving a total of 33 people, of whom 20 (60.61%; 95% CI 42.14–77.09) were women and 13 (39.39%; 95% CI 22.91–57.86) were men. The ages of the participants ranged from 2 months to 55 years, with an average age of 11.75 years (SD, 12.73 years). Most of the individuals were within 5 years of each other (12/33; 37.50%, 95% CI 21.10–56.31), and most of the participants were students (60.61%). Most individuals (84.85%) had very low income (USD 168). Most individuals (32/33; 96.97%, 95% CI 84.24–99.92) had direct contact with the ground. A total of 81.81% of the participants lived or had close contact with at least one dog, 6.06% lived with two dogs, and 12.12% lived with four dogs. A total of 9.09% (3/33) of the participants had never received anthelmintic drugs, 60.61% (20/33) had taken anthelmintics more than a year ago, and 30.30% (10/33) had received an anthelmintic in less than a year before the time of the interview (Table S1; supplementary data).

#### Clinical characteristics of humans

A history of asthma and arterial hypertension was observed in 6.06% (2/33; 95% CI 51.29–84.41) of the human participants, followed by a history of acute bronchiolitis, anemia, autism, arterial hypertension, cystitis, or pyelonephritis in 3.03% (1/33; 95% CI 0.08–15.76) of the participants. A history of prematurity was noted in one patient (3.03%; 1/33; 95% CI 0.08–15.76) and two women in the second trimester of pregnancy (6.06% (2/33; 95% CI 51.29–84.41)). A total of 28.13% (9/33) of the participants were undernourished, and 25.00% (8/33) were at risk of undernutrition (8/33) (Table S1).

#### Identification of intestinal parasites in humans

A total of 60.97% (23/33) of the human participants tested positive for intestinal parasites. The helminths identified by coprology were *Enterobius vermicularis* (12.12%), *Ascaris lumbricoides* (12.12%), *Ancylostoma* spp. (6.06%), and *Trichuris trichiura* (6.06%). Helminths were not found in 60.61% of the examined samples. A 6-month-old patient (3.03%) in close contact with a dog identified with *Toxocara* spp. had cutaneous larva migrans. The protozoa included *Giardia* spp. (15.15%), *Entamoeba histolytica/dispar* (12.12%), and *Blastocystis hominis* (3.03%). In the case of polyparasitism, *Ancylostoma* and *Giardia* spp. and *A. lumbricoides* and *E. histolytica/dispar* were found in 3.03% of the participants. The findings by age group revealed that helminths were dominated by *E. vermicularis* (15.38%) and *A. lumbricoides* (7.69%) and protozoa such as *Giardia* spp. (23.08%) in children under 5 years of age. In those aged 6–12 years, *Ancylostoma* spp. (9.09%) was found as a unique parasite and in parasitism with *Giardia* spp. The prevalence of other helminths, such as *T. trichiura*, was 9.09%. These helminths were also found in the group aged 19–35 years (Figure S4 and Table S1).

#### Clinical manifestations in humans

The general symptoms of the patients were loss of appetite (24.24%) and cough (21.21%) (p = 0.0928). Lack of appetite was the most common symptom in participants who were identified as having helminth parasites (30.77%; p = 0.0275). Regarding the global findings on physical examination, the most common symptoms were weight loss (33.33%), impetiginous skin lesions (15.15%), and severe dermatitis (6.06%). In patients infected with *Ancylostoma* spp., a predominance of impetiginous skin lesions (50%) and serpentine dermatitis (serpiginous eruption) (50%) was also found in patients who had cutaneous migrans larvae in close contact with a dog infected with *Toxocara* spp. (p = 0.5748). In patients infected with protozoa, weight loss was predominant (58.33%), especially in those infected with *Giardia* spp. (57.14%; p = 0.3081) (Figure S5b and Table S1).

### Animal health axis

#### Canine sociodemographic characteristics

Sixty-one canines were included. The owners of 53 of the samples agreed to participate in the study, of which 42 provided samples for the required laboratory analysis. A total of 45.24% (19/42; 95% CI 29.85–61.33) were females, and 54.76% were males (23/42; 95% CI 38.67–70.15). The most common age group was young adults (18 [42.86%], 95% CI 27.72–59.04), followed by adults (15 [35.71%], 95% CI 21.55–51.97), and finally puppies (9 [21.43%], 95% CI 10.30–36.81) (Figure S6a and Table S1). Most of the dogs were mixed breeds (73.81%; 95% CI, 57.96–86.14), followed by Fila Brasileiro (7.14%; 95% CI, 1.50–19.48), Pinscher (7.14%; 95% CI, 1.50–19.48), Yorkie (7.14%; 95% CI, 1.50–19.48), Labrador (2.38%; 95% CI, 0.06–12.57), and Pitbull (2.38%; 95% CI, 0.06–12.57). Most of the dogs had direct contact with the ground (90.24%: 95% CI, 76.87–97.28) (Table S1).

#### Canine clinical features

Food and nutritional status

Most of the dogs had a mixed diet consisting of commercial products and a family diet (27/42; 64.29%), followed by a family food supply (9/42; 21.43%), milk supplements (4/42; 9.52%), and food waste (2/42; 4.76%). A total of 11.90% (95% CI 3.98–25.63) of the adults had poor nutritional status (Figure S6b and Table S1).

Disease history, deworming, and rabies vaccination

Of the dogs, 7.14% (3/42) had a history of unclassified anemia, followed by 2.38% of those with urinary infection and obesity (Table S1). The results showed that 28.57% of the dogs had not received a rabies vaccination. A total of 43.9% had never been dewormed (Table S1).

#### Intestinal parasites in the canines

Intestinal parasites were identified in the stool samples of 33.33% (14/42) of the dogs included in the study. The most frequent unique parasite within helminths was *Ancylostoma* spp. at 14.29% (95% CI 5.43–28.54), followed by *Toxocara* spp. at 2.38% (95% CI 0.06–12.57). Among the protozoa, *Giardia* spp. were present in 7.14% (95% CI 1.50–19.48) of the samples. Co-infection was found with *Ancylostoma* spp. and *Giardia* spp., *Trichuris* spp. and *Giardia* spp., *Strongyloides* spp., and *Balantidium coli* in 2.38% (95% CI 0.06–12.57) of the samples, and polyparasitism with *Trichuris* spp., *A. lumbricoides*, and *Cystoisospora canis* in 2.38% (95% CI 0.06–12.57) of the samples. In puppies, *Toxocara* spp. and *Ancylostoma* spp./*Giardia* spp. co-infection (11.11% each) were observed. *Ancylostoma* spp. (11.11%), *Giardia* spp. (5.56%), co-infection with *Strongyloides* spp./*B. coli*, and polyparasitism with *Trichuris* spp./*A. lumbricoides*/*C. canis* (5.56% each) were observed in young adults. *Ancylostoma* spp. (26.67%), *Giardia* spp. (13.33%), and *Trichuris* spp./*Giardia* spp. co-infection were identified in adult dogs (6.67%) (p = 0.2627) (Figure S7a and Table S1).

#### Nutritional status and intestinal parasite types

Poor nutritional conditions were observed in dogs infected with *Ancylostoma* spp. (33.33% as a single type) and both *Trichuris* spp. and *Giardia* spp. (100%) (p = 0.046) (Figure S7b and Table S1).

#### Clinical manifestations and canine blood profile

**Figure 2:**
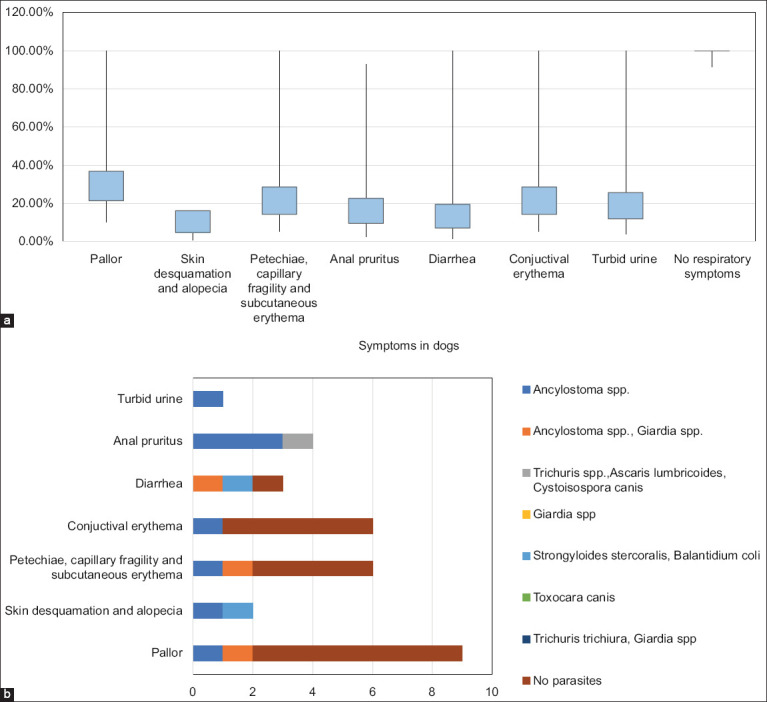
Clinical manifestations of parasitic infections in dogs. (a) Pallor and skin manifestations were the most frequent symptoms and signs in canines, among which were signs of alterations in hemostasis (petechiae, capillary fragility). (b) Clinical signs included gastrointestinal and dermatological abnormalities. Dogs infected with *Ancylostoma* spp. presented the greatest diversity in clinical presentation. *Toxocara* spp. was identified in one dog that did not present with any symptoms.

The symptoms and signs found in the canines were associated with the skin [desquamation and alopecia (4.76% CI 95% 0.58–16.16%); petechiae, capillary fragility, and subcutaneous erythema (14.29% CI 95% 5.43–28.54%); gastrointestinal (anal itching [9.52% CI 95% 2.66–22.62%]; diarrhea [7.14% CI 95% 1.50–19.48%]); ophthalmological (conjunctival erythema [14.29% CI 95% 5.43–28.54%]); urinals (urinary turbidity [11.90% CI, 3.98%–25.63%]); and pallor (21.43% CI 95% 10.30–36.81%) (Figure 2a and Table S1). Regarding the type of intestinal parasites, those infected with *Ancylostoma* spp. (11.11%) alone or together with *Giardia* spp. (11.11%) had the most severe pallor. Dermatological pathologies, such as desquamation and alopecia, were observed more frequently in canines infected with *Ancylostoma* spp. (50%) and *Strongyloides* spp./*B. coli* (50%). Some skin signs, such as petechiae, capillary fragility, and subcutaneous erythema, that could be related to capillary endothelial damage and vasculitis were found in two dogs infected with *Ancylostoma* spp. alone or together with *Giardia* spp. (16.6% each). Anal itching in canines occurred mainly in those with helminthiasis caused by *Ancylostoma* spp. (75%) or *A. lumbricoides*/*Trichuris* spp./*C. canis* (25%). Conjunctival erythema and turbidity in the urine (16.6% each) were detected in a dog with *Ancylostoma* spp. infection (Figure 2b and Table S1).

A blood test was conducted in 22 (52.38%) dogs. The average hemoglobin level in grams/dL (Hb g/dL) was 13.34 (SD 2.75) g/dL. There was no significant difference in hemoglobin values between purebred dogs (Brazilian line, Labrador, Pinscher, and Pitbull) and mixed-breed dogs (p = 0.4815). The average leukocyte count was 13.99 (SD 2.75)/10^9^/L. Compared with purebred dogs, mixed-breed dogs had higher leukocyte counts (x̅ 15.06 SD 4.19/10^9^/L), although the difference was not statistically significant (p = 0.2721). The average platelet count was 139 (SD 77.96) cells × 10^9^/L. The puppies had a lower average hemoglobin level of 11.15 (SD 2.25) g/dL, higher numbers of leukocytes (x̅ 18.40 SD 5.18 cells × 10^9^/L; p = 0.06376), and a lower number of platelets (x̅ 99 SD 35.53 cells × 10^9^/L; p = 0.2797) than the other age groups (Table S1).

#### Identification of Dirofilaria spp.

**Figure 3:**
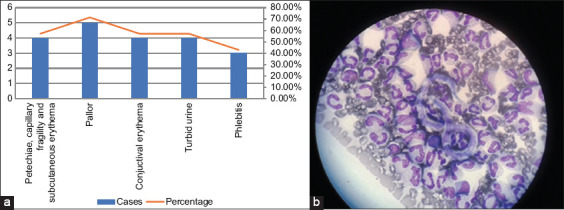
Clinical manifestations in dogs infected with *Dirofilaria* spp. (a) Dogs infected with *Dirofilaria* spp. presented with hematological alterations and vascular fragility, manifested mainly by cutaneous, ophthalmological, and renal symptoms. (b) During the evaluation of the canines, the veterinarians identified clinical signs suggestive of *Dirofilaria* spp. infection in some of the canines, for which they proceeded to perform the Woo technique or hematocrit centrifugation [11]. Blood samples were collected from the dogs in tubes containing di-ethylenediaminetetraacetate and potassium. Microhematocrit from samples prepared in single 75 mm × 1.2 mm capillary tubes were examined after centrifugation for 4 min in a microhematocrit centrifuge. The technique is based on the density difference between erythrocytes and plasma. When a blood sample in a capillary tube is centrifuged, the erythrocytes are grouped in the lower part of the tube because of their relatively high density, whereas the plasma and the intermediate layer (buffy coat), which contains leukocytes and platelets, are separated and located in a top layer, as are the extracellular parasitic forms that may affect the host. Each tube was placed horizontally on a clean microscope slide for examination, and the “thick layer” section of the hematocrit was focused after adjustment of the bottom stage condenser. An 8× eyepiece and a 45× objective were used. The presence of microfilariae was verified indirectly through the movement of erythrocytes caused by serpentine movements characteristic of these pathogens. Thin and thick smears were made on slides by depositing 10 μL of blood from the samples and staining with Giemsa (Reagen) that was spread over the surface of the slides to visualize the microfilariae, which were morphologically similar to *Dirofilaria* spp.

Seven (16.6%) participants tested positive for *Dirofilaria* spp. The affected dogs were adults and young adults (57.14% and 42.86%, respectively) (p = 0.4285). Dogs with *Dirofilaria* spp. infection presented with pallor (71.43% CI 95% 29.04–96.33), petechiae, capillary fragility, and subcutaneous erythema (57.14% CI 95% 18.41–90.10). Conjunctival erythema (57.14%; 95% CI, 18.41–90.10), phlebitis in skin puncture areas (42.86%; 95% CI, 9.90–81.59), and urinary turbidity (57.14%; 95% CI, 18.41–90.10) were observed (Figure 3). A lower average hemoglobin concentration was detected in these dogs than in dogs without *Dirofilaria* spp. infection (x̅ 12.5 g/dL (SD 3.0); x̅ 13.72 [SD 2.6], respectively; p = 0.3571) (Table S1) [10].

### Cross-species findings and risk factors

#### Intestinal parasitosis in dogs and humans

**Figure 4:**
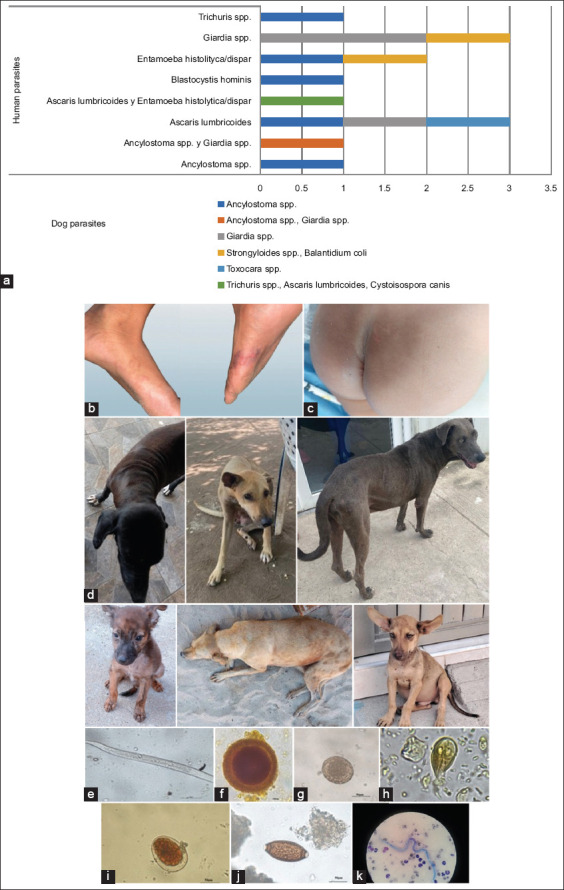
Clinical manifestations and intestinal parasites in soil and in close contact with humans and dogs. (a) *Protozoa*, such as *Giardia*, and helminths, such as *Ancylostoma* spp., were found in both species that share habitats or have constant close contact. (b) Cutaneous larva migrans were observed in children exposed to *Toxocar*a spp. and *Ancylostom*a spp., as seen on the feet of two school-age children. (c) On the buttocks of an infant. (d) Skin lesions (subcutaneous nodules, erythema, and alopecia) and nutritional deficiencies were observed in the dogs. (e) *Strongyloides* spp. rhabditiform larvae (40× objective). (f) *Toxocar*a spp. In human stool samples. (g) *A. lumbricoide*s eggs were found (40× objective). (h) *Giardi*a spp. trophozoites (40× objective). (i) Eggs from the family *Ancylostomatida*e. (j) *Trichuri*s spp. were identified in dogs. (k) *Dirofilari*a spp., an important zoonosis transmitted by local vectors such as *Aedes aegypt*i, was identified in 16.6% of dogs.

The common helminth species as a single type of parasite were *Ancylostoma* spp., *A. lumbricoides* spp., and *Toxocara* spp. (3.03% each). The most common protozoa were *Giardia* spp. (6.06%). One human participant shared common *Giardia* spp. and *Ancylostoma* spp. species (3.03%) with his dog (Figures 4 and S8).

#### Multivariable analysis of risk

In the logistic regression analysis, only the identification of helminths in dogs was observed as a statistically significant risk factor for the presence of parasitosis in humans (OR 0.105; CI 95% 0.014–0.808; p = 0.031) (Table S2). Dogs without deworming were at greater risk of parasites (OR 3.80; CI9 5% 0.984–14.66; p = 0.048) (Table S3).

## DISCUSSION

### Global burden of intestinal parasitic infections

Intestinal parasitosis is common in medium- and low-income countries. Soil-transmitted helminth infections are present throughout the Americas. According to the World Health Organization, approximately 46 million children aged between 1 and 14 years are at risk of being infected by parasites such as helminths, and the countries with the greatest incidence rates of parasitic infections are Brazil, Colombia, Mexico, Bolivia, Guatemala, Haiti, Honduras, Nicaragua, Peru, and the Dominican Republic [21].

### Evidence from the United States and Colombia

Moreover, in the United States, a study of free transit of dogs without a leash was conducted in 20 parks, and at least one intestinal parasite was found in 622 (20.7%) samples through the analyses of coproantigens by immunoassay and stool samples by centrifugal flotation with zinc sulfate, the most frequent being *Giardia* spp., *A. caninum* and *T. vulpis* [22].

A study was conducted in Las Guacas, Cauca, Colombia, to identify helminths and protozoa using microscopy and quantitative polymerase chain reaction (qPCR) in samples obtained from humans, their pets (dogs), and community water sources. Microscopic evaluation identified *Blastocystis* spp. (20.2%), *Endolimax nana* (5.6%), *Giardia* spp. (4%), *E. coli* (9.6%), and hookworm eggs (0.8%). The frequency of the different parasites detected by qPCR was higher (*Blastocystis* 87%, *A. duodenale* 22%, *Ancylostoma ceylanicum* 5.6%, *Giardia* 16%, *E. histolytica* 4.8%, *Cryptosporidium* spp. 9.6%, and *T. solium* 0.8%]. Among humans, the most common species were *Blastocystis* (90.1%), *Giardia* (3.1%), and *Ancylostoma* spp. (9.1%). The most common parasites identified in canines were *Ancylostoma* and *Blastocystis*, each accounting for 79% [23].

In Colombia, the prevalence of intestinal parasitosis was also determined in dogs from two animal welfare centers in Medellín and the eastern region of Antioquia. Diagnosis was made by direct examination with 0.8% saline and Lugol’s solution and the sheath flotation method. The number of enteroparasites was 72.1%. Eleven parasitic agents were identified, of which the most prevalent were *Uncinaria stenocephala*, with 39.7%; *A. caninum*, with 20.6%; *T. vulpis*, with 16.2%; and *Toxocara* spp., with 11.8% [24].

### Littoral regions and environmental drivers

Important zoonotic intestinal parasites have been identified in littoral regions, which has led to the need for sanitary interventions to reduce the risk of disease transmission by these parasites [25]. In Ilhéus, Brazil, the climatic conditions, interaction between humans and dogs, and contact with synanthropic animals and insects in garbage dumps favor the proliferation and spread of intestinal parasites. Other factors that influence this region include the lack of adequate garbage disposal services and the large number of common areas that enable the interaction of infected and uninfected individuals and animals [26].

### One Health findings from coastal Cartagena (current study)

This study was conducted in coastal populations in Cartagena, Colombia, and analyzed using the One Health approach, important data on intestinal parasites in humans and canines were obtained, many of which were free-roaming and in direct contact with one another. Moreover, the environmental study revealed contamination of soils with intestinal parasites from dog feces. The environmental health team has been analyzing various coastal areas in Cartagena, Colombia, and has identified parasites, such as *Toxocara* spp. (50.2%), *Ancylostoma* spp. (25.5%), and *Strongyloides* spp. (24.28%), more frequently in dog feces. This group found a greater abundance of *Strongyloides* spp. and *Toxocara* in the dry season, whereas the abundance of *Ancylostoma* spp. was greater in the rainy season (14.8%).

### Comparative coastal evidence (Ecuador and Brazil)

Studies of the feces of dogs that roam freely on public beaches in coastal areas of Ecuador revealed greater frequencies of *Ancylostoma* spp. (19.4%) and *Toxocara* spp. (7.2%). They also observed *Trichuris* spp., *D. caninum, Diphyllobothrium* spp., *Capillaria* spp., *Dicrocoelium* spp., *Heterobilharzia americana, Hymenolepis* spp., and *Spirocerca* spp. The authors reported a statistically significant difference between the humid and dry tropical ecoregions (p = 0.0102), with a high prevalence of 35.7% (27.3–44.1, 95% CI) in the humid ecoregion. Similar to the findings from the coastal regions of Cartagena, Colombia, the parasite *Ancylostoma* spp. presented a significantly higher prevalence in the humid region (p < 0.05) (26).

### Local risk factors in La Boquilla and Punta Arenas

These factors are also present in Punta Arenas and La Boquilla, where the scarce hygienic and environmental conditions observed in many of the areas examined may favor the development and transmission of zoonotic parasitic diseases. This risk is increased because 51.52% of the participants in this study live with one dog, 12.12% live with four dogs, and most of the canines have direct contact with the ground (90.24%; 95% CI, 76.87–97.28). Additionally, 43.9% have never been dewormed. Moreover, 16.67% and 9.09% of humans, especially those under five years of age and those in the school stage group (6–12 years), respectively, had never been dewormed. Most participants had little economic income, which could have contributed to malnutrition (28.13%) and related symptoms (25.00%), which could be both a risk factor and a consequence of intestinal parasitic infection.

### Human–canine parasitic profiles and shared infections

A total of 60.97% of the human participants were positive for intestinal infection with helminths, such as *E. vermicularis*, *A. lumbricoides*, and *T. trichiura*, and protozoa, such as *Giardia* spp., *E. histolytica/dispar*, and *B. hominis*. It was important to identify *Ancylostoma* spp. and *T. canis*, which are usually the most infectious helminths in canines. The identification of common intestinal parasites among humans living with or in close contact with dogs was interesting, as *Ancylostoma* and *T. canis* spp. were found, in addition to *A. lumbricoides* and *Giardia* spp. A human participant shared common *Giardia* spp. and *Ancylostoma* spp. species with his companion dog.

### Corroborating evidence from Chile and Mexico

In close contact with humans and dogs, intestinal parasites have also been reported in Santiago de Chile, Chile [27], in immunocompromised children and their pets (35 dogs and 9 cats), and the parasites *Giardia intestinalis* (8.57%), *T. vulpis* (11.42%), and *T. canis* (2.85%) were found in dogs. Two children and their respective pets were diagnosed with the same zoonotic diseases (toxocariasis and giardiasis). In their study, they confirmed that the patients’ domestic dogs and cats represented a reservoir of potentially serious zoonotic infections, which was intensified by the lack of routinely recommended vaccines and antiparasitics. Although the protective mechanism of the rabies vaccine against other pathologies is not well understood, the possible biological mechanisms responsible for the non-specific reactions of the vaccines have been reviewed. These include the cross-reactivity of T lymphocytes or heterologous immunity, as well as the epigenetic reprogramming of innate immune cells, known as innate trained immunity [28–30]. A Chilean study revealed that arthropods are potential vectors of zoonotic infections in 49% of dogs and 44% of cats (28). The serological presence of *T. canis* (29.2%), *Toxoplasma gondii* (91%), and *Trichinella spiralis* (6.7%) has also been reported in humans in other regions, such as Yucatán, Mexico. A total of 46.2% of the fecal samples from the dogs contained gastrointestinal parasites, with 12% of these samples containing *T. canis*.

### Clinical correlates and pediatric vulnerability

In humans with protozoa infections, especially those caused by *Giardia* spp., weight loss was the predominant symptom (p = 0.3081), whereas impetiginous skin lesions and serpiginous dermatitis were observed in humans infected with *Ancylostoma* spp. and *T. canis* (p = 0.5748). Although these observations are not statistically significant (which could be attributed to the small sample size), the clinical signs of pallor and dermatological lesions found in dogs may reflect pathologies associated with endothelial, infectious, and nutritional alterations, especially in those infected with *Ancylostoma* spp., *T. canis*, and S. stercoralis. Considering that these parasites have larval stages and are involved in the etiology of cutaneous larva migrans, they should be considered in the diagnosis of impetiginous and serpiginous skin manifestations in pediatric patients, similar to the clinical cases of infection by *Ancylostoma* spp. and *T. canis* (Figure 4). A similar situation was described in the community of Milagro, Guayas, Ecuador, in which *Ancylostoma* spp. caused cutaneous larva migration on the sole of the foot of a 10-year-old boy [31]. Pediatric patients have a greater risk of *T. canis* infection due to a lower awareness of health prevention and certain habits, such as frequent hand–mouth contact and geophagy [32]. Toxocariasis is an understudied disease and a threat to public health with high economic impact and prevalence among children from socioeconomically disadvantaged populations, both in the tropics and subtropics [33].

### Vector-borne co-infections and regional surveillance

Moreover, in these regions, there are also other aggravating factors, such as the presence of vectors such as *Aedes aegypti* [34]. In La Boquilla and Punta Arenas, mosquitoes can also carry diseases [35] such as *Dirofilaria*sis because these nematodes were detected in 16.6% of the dogs who were subjected to a Woo test under suspicion of possible infection with microfilariae due to the presence of cutaneous signs that could be related to capillary endothelial damage and vasculitis. *Dirofilaria immitis is* the causal agent of cardiopulmonary heartworm disease in dogs and cats and it can also infect humans. This study was conducted to determine the frequency and factors associated with *D. immitis* infection in pet dogs in the metropolitan area of the Colombian Caribbean (northern Colombia). A total of 173 dogs were analyzed by a commercial rapid immunochromatographic test (RIT) and a nested polymerase chain reaction (PCR) of the cytochrome oxidase subunit I gene, in parallel. Ninety-two (53.2%) dogs showed positive results to the RIT, while 59 (34.1%) animals had *D. immitis* DNA by PCR. Positivity to one or both diagnostic techniques was detected in 104 (60.1%; CI 95%: 53.8–67.4) of the sampled dogs [36]. The presence of *D. immitis* in shelter dogs in Bucaramanga, Colombia They reported an overall prevalence of *D. immitis* of 6.3% (13/207) and 0.5% (1/207) by blood smear and the Knott test using an immunochromatography test kit, respectively, without significant differences according to sex, age group, dog breed, and coat length (p > 0.05) [37]. To monitor vector-borne diseases in dogs in the cities of Medellín, Barranquilla, and Cartagena in Colombia and determine the existence of diseases such as cardiopulmonary heartworm caused by *D. immitis*, ehrlichiosis by *Ehrlichia canis*, Lyme disease by *Borrelia burgdorferi*, and anaplasmosis by *Anaplasma phagocytophilum*, a rapid test system was performed using the enzyme-linked immunosorbent assay, which revealed a prevalence of E. canis, *A. phagocytophilum*, *D. immitis*, and *B. burgdorferi* of 62%, 33%, 1.2%, and 0%, respectively. In Medellín, the rates were 26% for E. canis, 12% for *A. phagocytophilum*, and 0% for *D. immitis*. In Barranquilla (neighboring city of Cartagena, Colombia), the prevalence rates of E. canis, *A. phagocytophilum*, and *D. immitis* were 83%, 40%, and 2%, respectively. In Cartagena, the percentages were 80%, 51%, and 3% for *E. canis*, *A. phagocytophilum*, and *D. immitis*, respectively [38].

Previous studies conducted in Barranquilla and Puerto Colombia (geographically close) in Colombia reported a prevalence of 61.86% for *Ehrlichia* spp., followed by 22.03% for *Anaplasma* spp., 11.30% for *D. immitis*, and 0.56% for *B. burgdorferi* [39–41].

## CONCLUSION

This study revealed a substantial presence of zoonotic intestinal parasites in humans, dogs, and the environment along the coastal area of Cartagena. Among 78 soil samples, 50.7% were positive for one or more parasites, with *Toxocara* spp. (46.2%), *Strongyloides* spp. (28%), and *Ancylostoma* spp. (25.7%) being most prevalent. Seasonal variation was evident, with *Toxocara* and *Strongyloides* more abundant in the dry season, and *Ancylostoma* peaking during the rainy season. In humans, 60.97% were positive for intestinal parasitosis, with protozoa such as *Giardia* spp. (15.15%) and *E. histolytica/dispar* (12.12%), and helminths including *E. vermicularis* and *A. lumbricoides* (12.12% each) were detected. Clinical manifestations such as loss of appetite, weight loss, cough, and skin lesions were particularly associated with helminth infections. Among dogs, 33.33% were infected with *Ancylostoma* spp. (14.29%), *Toxocara* spp. (2.38%), and *Giardia* spp. (7.14%) being most frequent, and coinfections were common. Notably, dogs with poor nutritional status were more likely to harbor parasites, and *Dirofilaria* spp. was detected in 16.6% of the sampled canines, causing clinical signs related to endothelial damage. Importantly, shared species such as *Giardia* and *Ancylostoma* were identified in human–dog pairs, and regression analysis showed that helminths in dogs were a significant risk factor for human parasitosis, with lack of canine deworming further increasing the risk.

These findings have practical implications, emphasizing the urgent need for integrated interventions such as synchronized human and canine deworming campaigns, promotion of veterinary preventive care, environmental sanitation measures, and targeted education on hygiene. Seasonal patterns suggest that control strategies should be adapted to suit specific climate conditions. The strength of this study lies in its One Health design, which integrates environmental, veterinary, and human health data in a neglected coastal setting, as well as in the seasonal and clinical insights it provides. However, limitations include the relatively small and convenience-based sample size, lack of molecular genotyping to confirm shared transmission pathways, and potential seasonal bias due to climatic variability.

Future research should apply molecular tools to track parasite transmission across hosts, conduct longitudinal and intervention-based studies, and expand surveillance to include vector-borne pathogens. This study highlights the high burden of zoonotic intestinal parasites in Cartagena’s coastal communities, particularly among socioeconomically disadvantaged groups, and underscores the necessity of coordinated One Health approaches to reduce transmission risks and improve community health.

## DATA AVAILABILITY

The supplementary data can be made available from the corresponding author upon request.

## AUTHORS’ CONTRIBUTIONS

DFR: Supervised the study. DFR, ITB, MMA, JMM, NLC, MMF, SPS, ABH, JGB, VQC, JPP, LCB, NVG, CCC, ASM, JFR, AHM, KNG, DSU, and MSM. Conceptualized and performed the study and drafted and revised the manuscript and DFR, JMM, and MSM: Performed statistical analysis. DFR, ITB, MMA, JMM, NLC, MMF, SPS, ABH, JGB, VQC, JPP, LCB, NVG, CCC, ASM, JFR, AHM, KNG, DSU, and MSM: Interpreted the results and drafted and revised the manuscript. All authors have read and approved the final manuscript.
